# Supplementary material for: PWWP2A binds distinct chromatin moieties and interacts with an MTA1-specific core NuRD complex
Source: Nat Commun. 2018 Oct 16;9:4300. doi: 10.1038/s41467-018-06665-5 (PMC6191444; doi:10.1038/s41467-018-06665-5)

# Supplementary information

## PWWP2A binds distinct chromatin moieties and interacts with an MTA1-specific core NuRD complex

Stephanie Link, Ramona M.M. Spitzer, Maryam Sana, Mario Torrado, Moritz C. Völker-Albert, Eva C. Keilhauer, Thomas Burgold, Sebastian Pünzeler, Jason K.K. Low, Ida Lindström, Andrea Nist, Catherine Regnard, Thorsten Stiewe, Brian Hendrich, Axel Imhof, Matthias Mann, Joel P. Mackay, Marek Bartkuhn and Sandra B. Hake

## Supplementary figures and legends

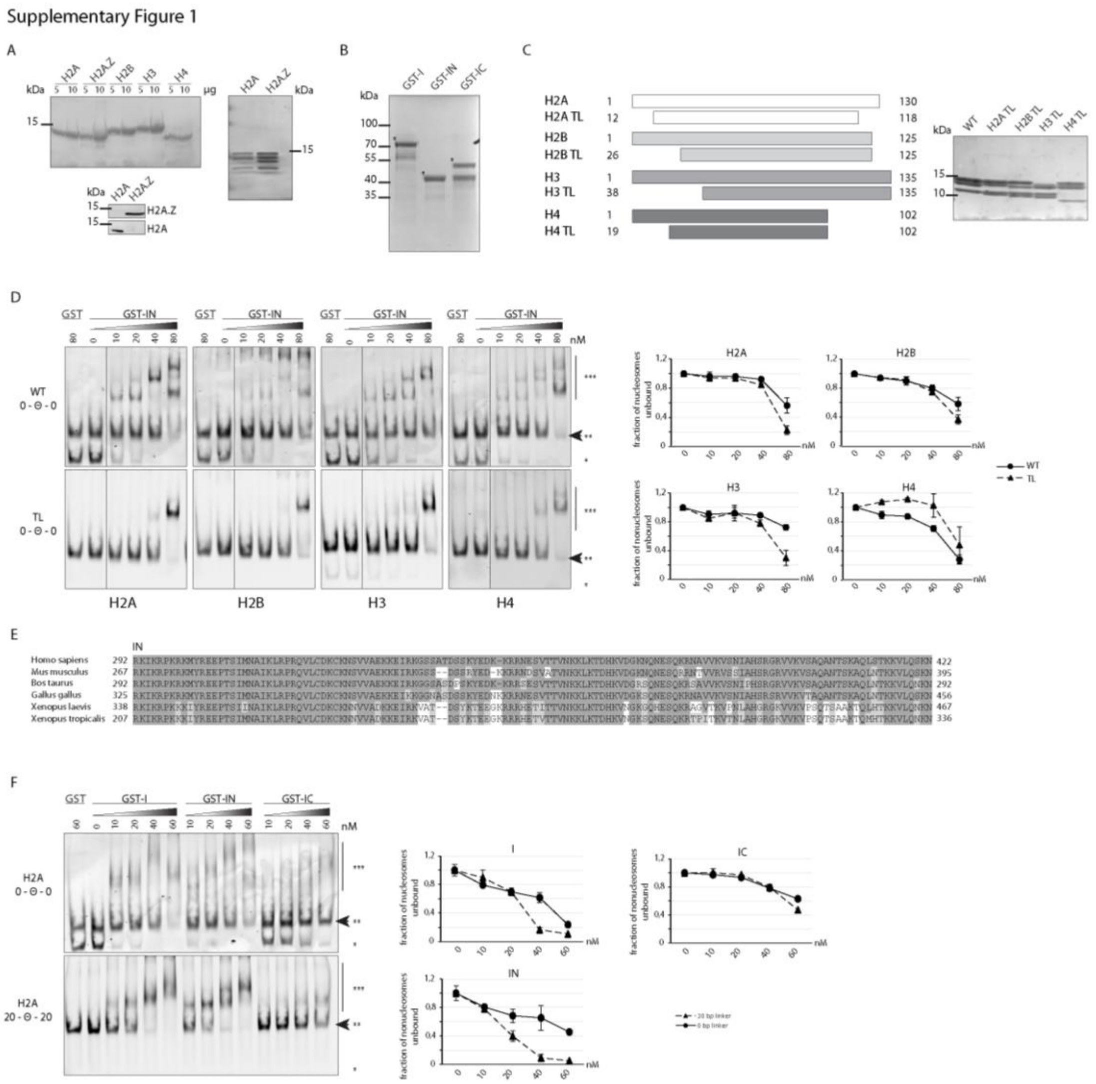


**Supplementary Figure 1: GST-IN binds independent of histone tails to recombinant mononucleosomes**

**(a)** Top: Coomassie-stained SDS-PAGE of purified canonical recombinant histones and histone variant H2A.Z (left) and H2A- or H2A.Z-containing octamers (right). Bottom: Immunoblotting of SDS-PAGE separated octamers with H2A- or H2A.Z-specific antibodies confirms the exclusive presence of the respective incorporated variant.

**(b)** Coomassie-stained SDS-PAGE of purified recombinant GST-I, GST-IN and GST-IC proteins. *: indicates bands at the predicted sizes for each protein. Other bands are, most likely, degradation products.

**(c)** Left: Schematic of different tail-less (TL) histone mutants in comparison to their respective wild-type counterparts. Right: Coomassie-stained SDS-PAGE of different recombinant TL histone-containing mononucleosomes.

**(d)** Left: Representative cEMSAs using different TL mutants. Recombinant mononucleosomes without linker DNA (0–Θ–0) and either WT histones (top) or TL histones (bottom) were incubated with the indicated concentrations of GST-IN.

Right: Quantification of signal intensities of nucleosomes (**) using Image Studio Lite Ver 5.2 (LI-COR). Error bars indicate SEM of three independent replicates.

**(e)** Amino acid sequence alignment of the IN region of PWWP2A from the indicated species. IN sequences of *Homo sapiens* ´Q96N64 [https://www.ncbi.nlm.nih.gov/protein/Q96N64/]´, *Mus musculus* ´Q69Z61 [https://www.ncbi.nlm.nih.gov/protein/Q69Z61]´, *Bos taurus* ´F1MKS1 [https://www.ncbi.nlm.nih.gov/protein/XP_024851055.1]´, *Gallus gallus* ´XP_015149148.1 [https://www.ncbi.nlm.nih.gov/protein/XP_015149148.1]´, *Xenopus laevis* ´XeXenL6RMv10002517m [https://www.uniprot.org/uniprot/A0A1L8GR68]´ and *Xenopus tropicalis* ´XM_002940175 [https://www.ncbi.nlm.nih.gov/protein/XP_002940221]´ were aligned using the MUSCLE multiple sequence alignment tool with default settings. Identical amino acids are highlighted in dark gray, similar amino acids in light gray and changes are set apart on a white background.

(**f**) Left: Representative cEMSA using recombinant H2A-containing mononucleosomes without (0–Θ–0, top) and with (20–Θ–20, bottom) linker DNA incubated with the indicated concentrations of GST-I, GST-IN and GST-IC. *free DNA, **nucleosome, ***nucleosome GST-protein complex. Arrow indicates loss of signal when nucleosome GST-protein complexes are formed.

Right: Quantification of signal intensities of nucleosomes (**) using Image Studio Lite Ver 5.2 (LI-COR). Error bars indicate SEM of two independent replicates.

Supplementary Figure 2

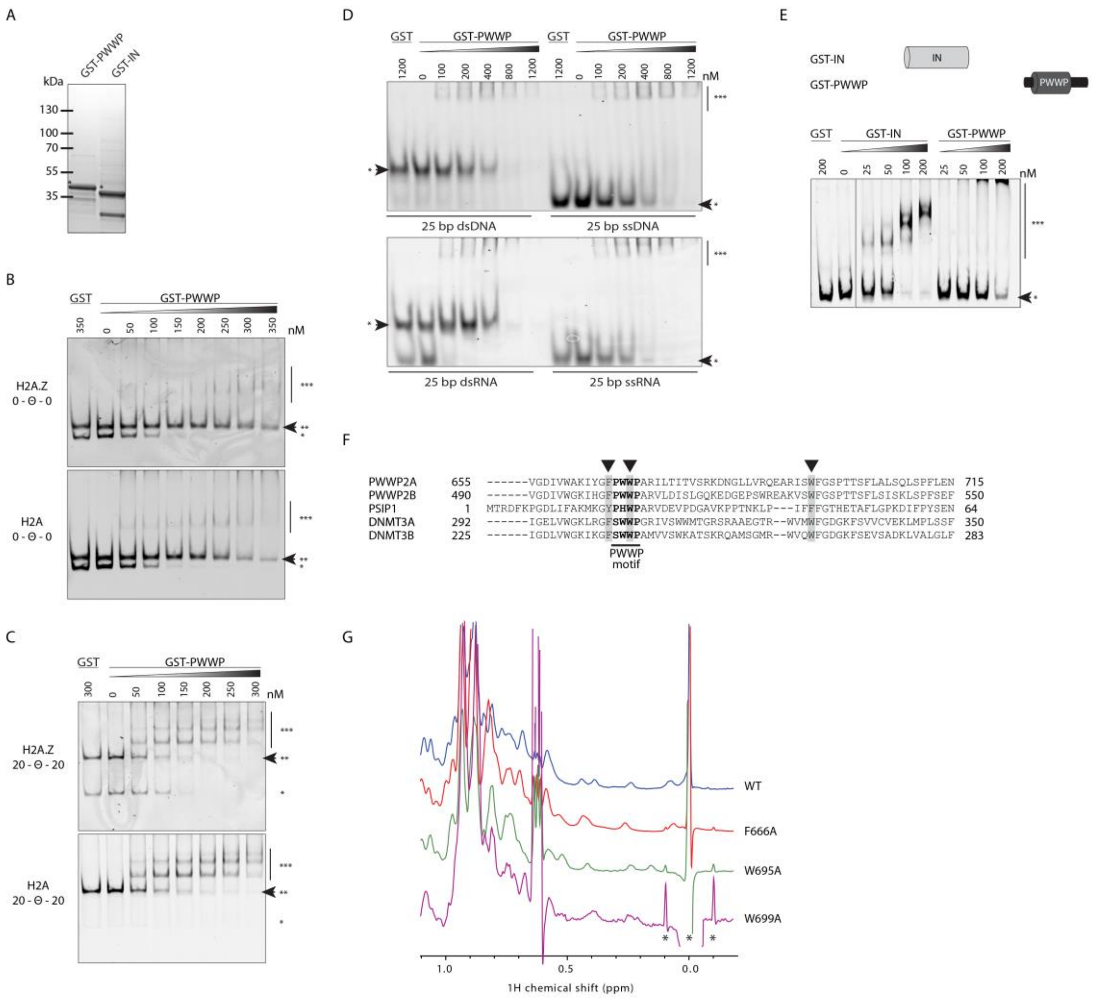


**Supplementary Figure 2: PWWP2A's PWWP domain does not distinguish between H2A and H2A.Z nucleosomes *in vitro* and interacts with nucleic acid strands in general**

**(a)** Coomassie-stained SDS-PAGE of purified recombinant GST-PWWP and GST-IN proteins. *: indicates bands at the predicted sizes for each protein. Other bands are, most likely, degradation products.

**(b)** Representative cEMSA using recombinant H2A.Z- (top) or H2A- (bottom) containing mononucleosomes without linker DNA (0–Θ–0) incubated with the indicated concentrations of GST-PWWP. GST alone served as negative control. *free DNA, **nucleosome, ***nucleosome GST-protein complex. Arrow indicates loss of signal when nucleosome GST-protein complexes are formed.

**(c)** same as in **b**, except for using mononucleosomes containing 20 bp linker DNA (20–Θ–20).

**(d)** Representative EMSAs using either ss or ds DNA and RNA with a length of 25 bp incubated with the indicated amounts of GST-PWWP. *free DNA or RNA, ***GST-PWWP-nucleic acid complex. Arrow indicates loss of signal when DNA-GST-PWWP complex is formed.

**(e)** Top: Schematic representation of recombinant GST-PWWP2A deletions (GST-IN, GST-PWWP) used in EMSAs. Bottom: Representative EMSA similar to (**d**) using Cy-5 labeled 187-bp dsDNA and the indicated concentrations of GST-PWWP2A deletions. *free DNA, ***DNA-GST-protein complex. Arrow indicates loss of signal when DNA-GST-protein complexes are formed.

**(f)** Sequence alignment of PWWP domain sequences of DNMT3-related PWWP domain containing proteins (PWWP2A ´Q96N64 [https://www.ncbi.nlm.nih.gov/protein/Q96N64/]´, PWWP2B ´Q6NUJ5 [https://www.ncbi.nlm.nih.gov/protein/Q6NUJ5]´, DNMT3A ´Q9Y6K1 [https://www.ncbi.nlm.nih.gov/protein/Q9Y6K1]´, DNMT3B ´Q9UBC3 [https://www.ncbi.nlm.nih.gov/protein/Q9UBC3]´ and PSIP1 ´O75475 [https://www.ncbi.nlm.nih.gov/protein/O75475]´) as defined in UniProt [ID]. Alignment was done using MUSCLE. Residues forming the aromatic cage are highlighted in gray and indicated with arrow heads.

**(g)** Methyl region of one-dimensional 1H NMR spectra of wild-type S-PWWP and of the indicated aromatic-cage mutants. The presence of signals between 0 and 0.5 ppm indicates that all three mutants form stable, well-defined folds.

Supplementary Figure 3

A

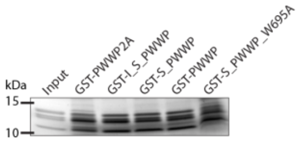


B

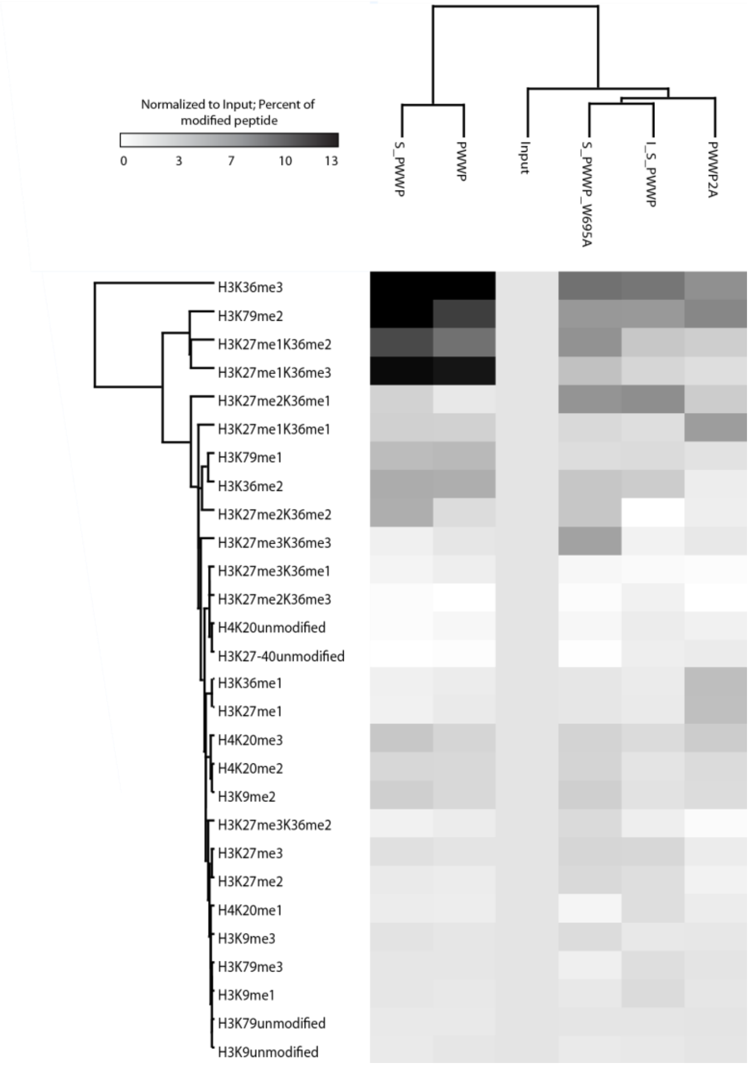


**Supplementary Figure 3: The PWWP domain of PWWP2A interacts with H3K36me3.**

**(a)** GST-tagged PWWP2A, I_S_PWWP, S_PWWP, PWWP and S_PWWP_W695A were incubated with HK cell-derived mononucleosomes, separated by SDS-PAGE and stained with Coomassie. Excised histone bands served as input for mass spectrometry.

**(b)** Heatmap of the abundance of various histone modifications from pull-down experiments with HK cell-derived mononucleosomes (see (**a**)), determined by mass spectrometry (two independent experiments) and normalized to input. Scale-bar: percentage of modified peptide identified.

Supplementary Figure 4

A

| Domain | DNA | H2A MN 0 - Θ - 0 | H2A MN 20 - Θ - 20 | H2A.Z MN 0 - Θ - 0 | H2A.Z MN 20 - Θ - 20 | TL MN 0 - Θ - 0 | TL MN 20 - Θ - 20 | H3K36me3 |
|---|---|---|---|---|---|---|---|---|
| I | ++ | + | ++ | + | +++ | n.d. | n.d. | - |
| IN | ++ | + | ++ | + | ++ | +* | ++** | n.d. |
| IC | ± | ± | ± | +++ | +++ | n.d. | n.d. | n.d. |
| PWWP | ± | ± | ++ | ± | ++ | n.d. | n.d. | - |
| S_PWWP | n.d. | n.d. | n.d. | n.d. | n.d. | n.d. | n.d. | +++ |
| PWWP2A_ΔIC | n.d. | n.d. | n.d. | n.d. | n.d. | n.d. | n.d. | +++ |

*except TL H4 (++)

**except TL H3 (+++)

B

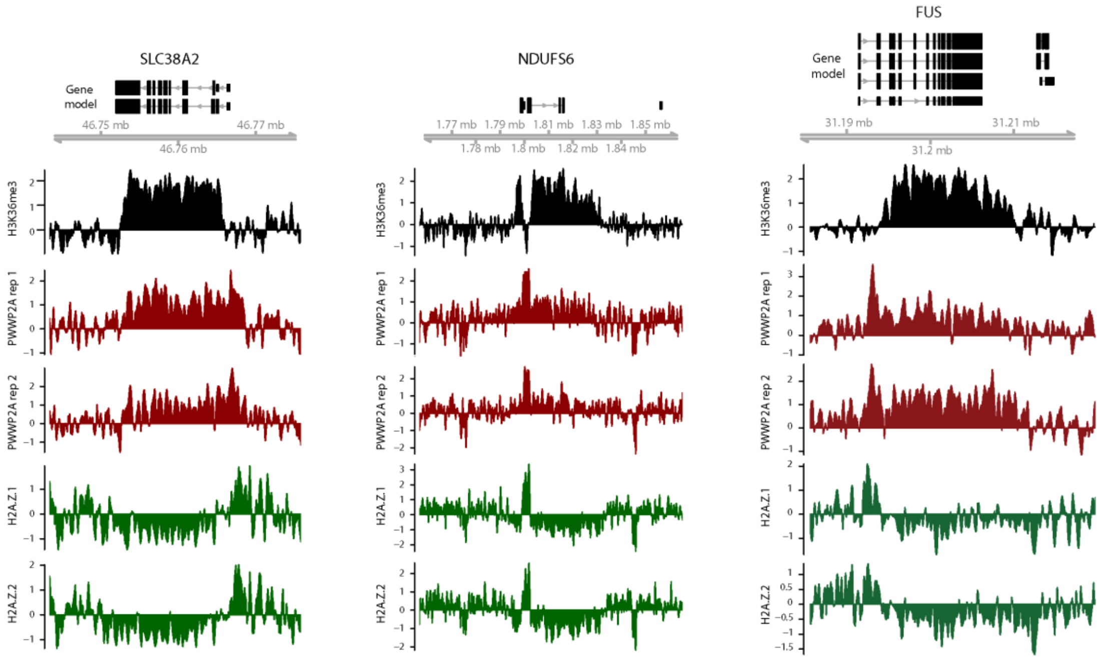


**Supplementary Figure 4: H3K36me3-positive gene body regions are weakly enriched in PWWP2A and depleted in H2A.Z.1 and H2A.Z.2.**

**(a)** Summary table illustrating PWWP2A's various binding modes to different chromatin moieties. MN: mononucleosomes, TL: tail-less, n.d.: not determined, -: no interaction observed, + to +++: weak to strong interactions observed.

**(b)** Genome browser snap shots of the human SLC38A2, NDUFS6 and FUS gene loci as representative regions displaying H3K36me3 (black), two GFP-PWWP2A replicates (red) and GFP-H2A.Z.1 and GFP-H2A.Z.2 (green) nChIP-seq signals. See also Fig. 3 for further data analysis.

Supplementary Figure 5

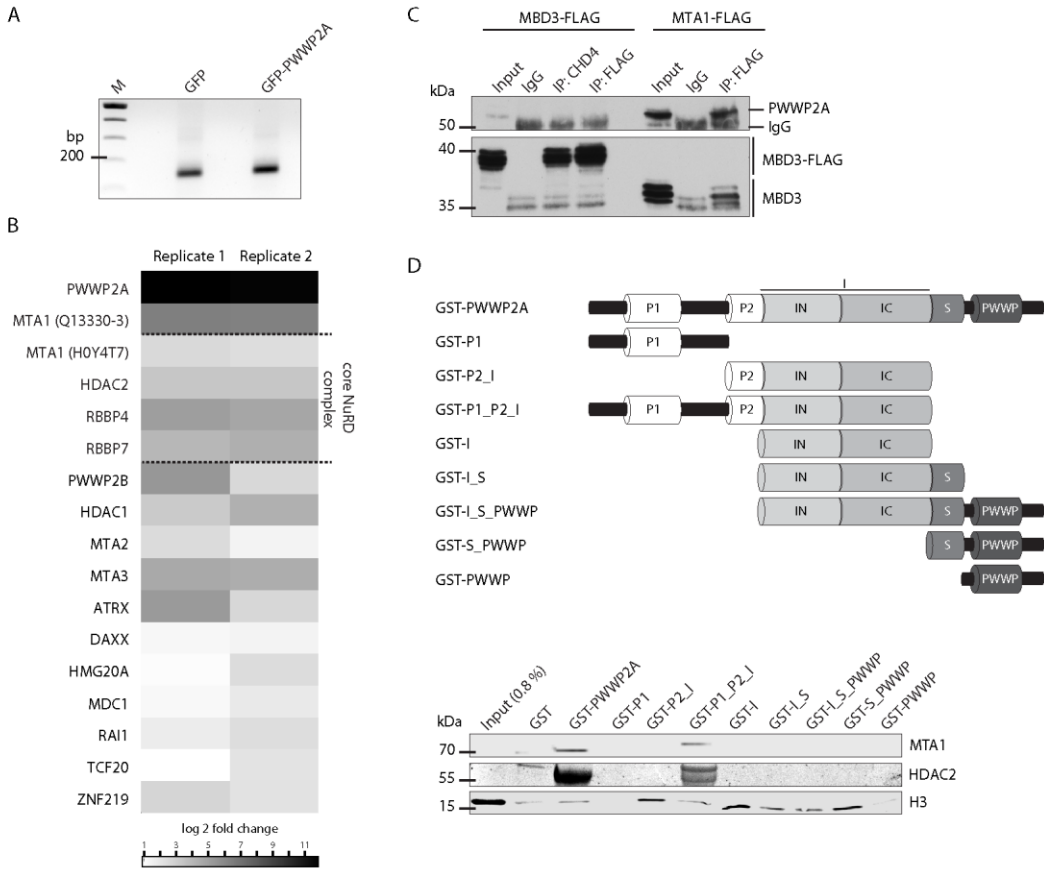


**Supplementary Figure 5: PWWP2A interacts with MTA1-specific core NuRD (M1HR) complex**

**(a)** Agarose gel illustrating MNase digest of cell-derived mononucleosomes from HK cells stably expressing GFP or GFP-PWWP2A serving as input for label-free qMS/MS.

**(b)** Hierarchical clustered and plotted heatmap of GFP and GFP-PWWP2A interacting proteins (n=2), calculated using Perseus software. Log2 LFQ intensities are presented (fold-change for GFP-PWWP2A vs GFP pulldowns) for the strongest GFP-PWWP2A binders.

(**c**) Immunoblot of PWWP2A and MBD3 after endogenously tagged MBD3-FLAG or MTA1-FLAG, CHD4 or control (IgG) IPs from mESC nuclear extracts. Input lanes represent 10% of the amount of lysate used for immunoprecipitation.

(**d**) Top: Schematic depiction of domain structure of PWWP2A and deletion constructs. Bottom: Immunoblots detecting endogenous MTA1, HDAC2 and H3 upon GST and distinct GST-PWWP2A truncation IPs using HK cell-derived mononucleosomes.

Supplementary Figure 6

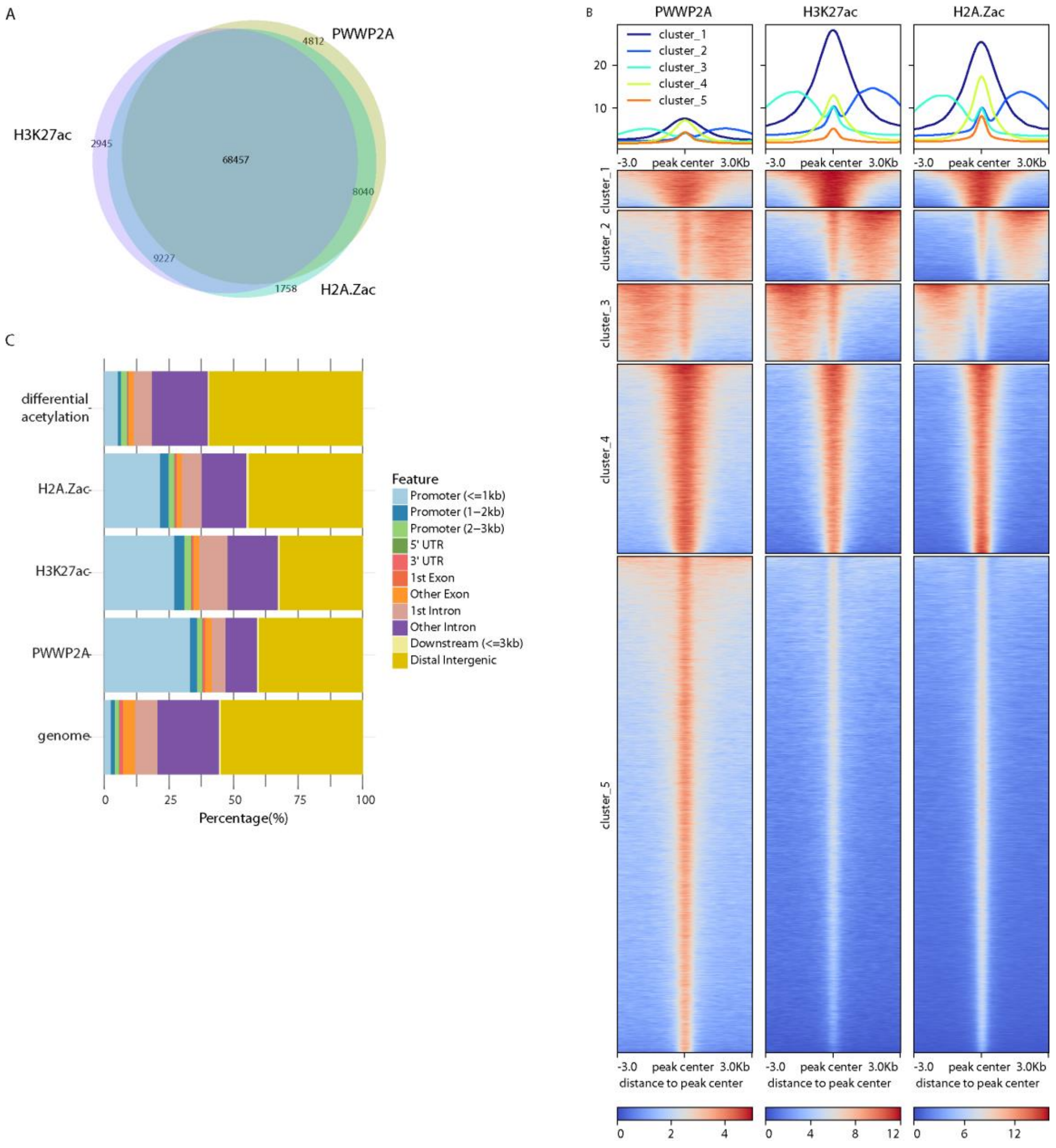


**Supplementary Figure 6: Relation of PWWP2A with H3K27ac and H2A.Zac**

(**a**) Venn diagram depicting overlap of genome-wide PWWP2A, H3K27ac and H2A.Zac sites. The overlap calculation followed a protocol described by Stielow et al.[1] and is described in the materials and methods section.

(**b**) Heatmap combining the peak sets of PWWP2A, H3K27ac and H2A.Zac into a unified set and collection of binding data in 6 kb spanning windows spanning the peak centers. In total, 98953 peaks were analyzed. Data was portioned into 5 clusters by k-means clustering. All three factors show highly similar binding patterns genome-wide.

(**c**) Feature distribution proportioning the hg19 genome into the indicated functional annotations based on UCSC hg19 gene annotations. Peak sets detected in H3K27ac, H2A.Zac and PWWP2A generally show a relatively broad spectrum of genomic annotations, but are strongly associated with promoter-associated annotations (light blue and green colors) as indicated by the comparison to the genomic background, where these annotations are only represented by a minor fraction of the genome. In contrast, the subset of H3K27ac sites induced after PWWP2A knockdown reveals a less specific distribution with the majority of peaks mapping to intergenic and intronic regions similar to the genomic background distribution.

Supplementary Figure 7

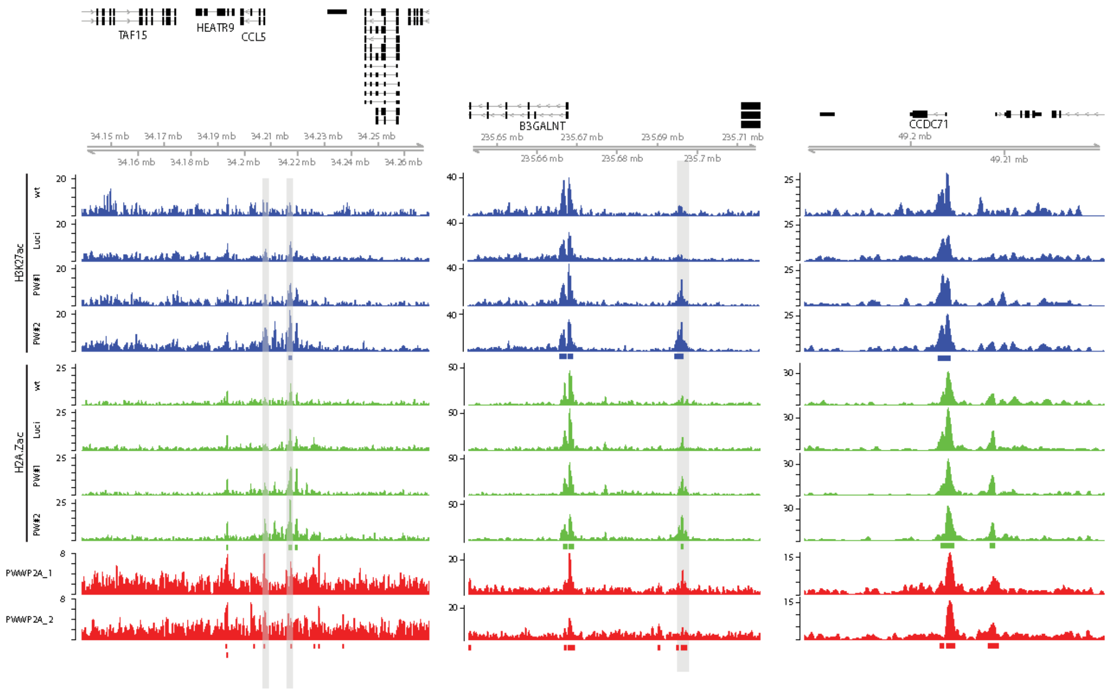


**Supplementary Figure 7: Depletion of PWWP2A leads to an increase of acetylation levels on H3K27 and H2A.Z**

Genome browser snap shots of three representative regions in the human genome displaying H3K27ac (from top to bottom: wt, Luci, PW#1, PW#2) (blue), H2A.Zac (wt, Luci, PW#1, PW#2) (green) and two GFP-PWWP2A replicates (red) nChIP-seq signals. Differentially acetylated sites are shaded in grey. Notice two different classes of genes/loci: Increase of acetylation levels on H3K27ac and H2A.Zac can result in (i) upregulation of gene expression of a nearby gene (CCL5, left)) or (ii) no change in gene expression of a nearby gene (B3GALTN2, middle); CCDC71 represents a gene/locus where both acetylation level and gene expression remain unchanged (right). See also Fig. 6 for further data analysis.

**Supplementary Data 1 (PRM inclusion list):**
List of precursor masses for specified histone modifications and their respective processing parameters for PRM analysis.

**Supplementary Data 2 and 3 (MaxQuant output Excel files):**
List of identified proteins of replicate 1 (Supplementary Data 2) and replicate 2 (Supplementary Data 3) of GFPPWWP2A mononucleosome IPs in label-free MS-based proteomics after MaxQuant analysis.

## Supplementary references

1. Stielow C, Stielow B, Finkernagel F, Scharfe M, Jarek M, Suske G. SUMOylation of the polycomb group protein L3MBTL2 facilitates repression of its target genes. *Nucleic Acids Res* **42**, 3044-3058 (2014).

Supplementary Figure 8: Uncropped blots for Figure 2

Figure 2C

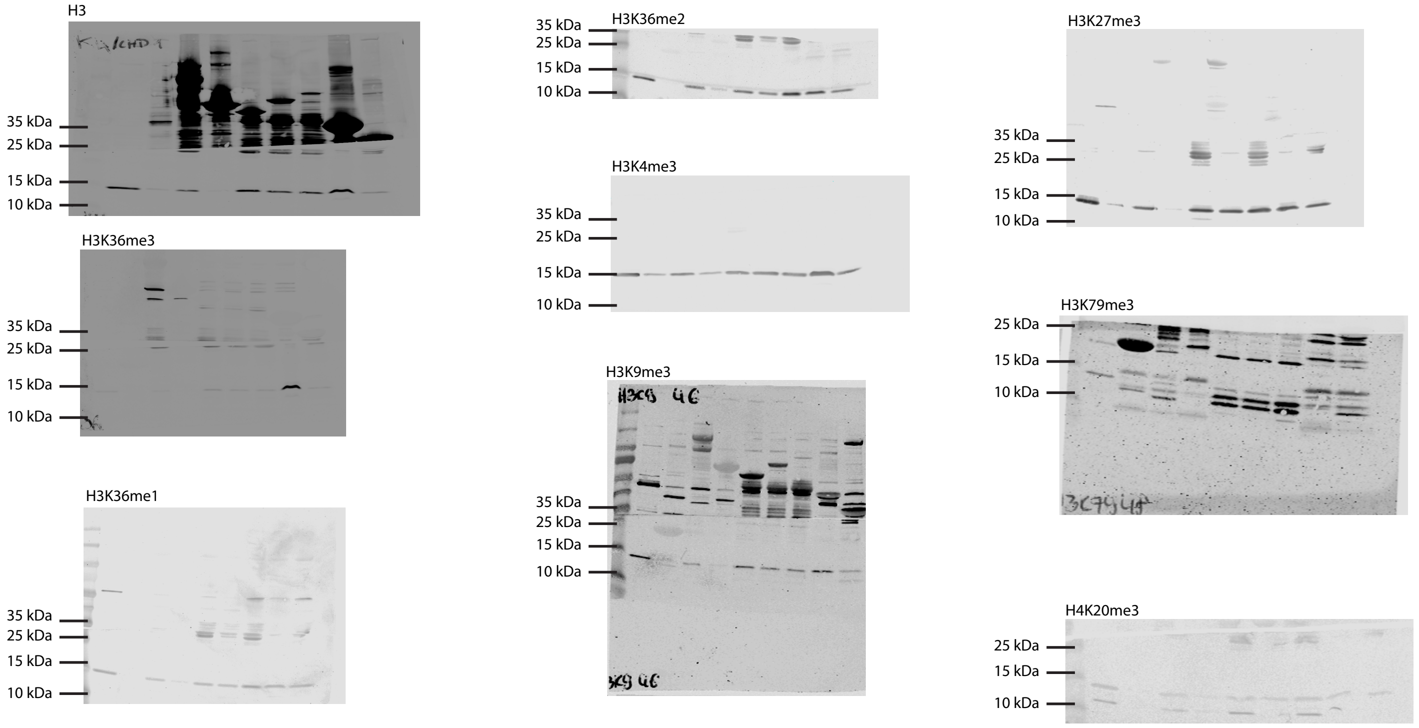


Figure 2D

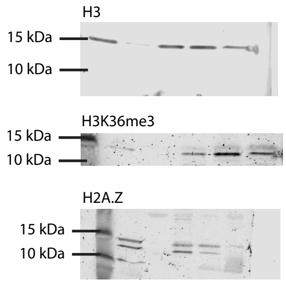


Figure 2E

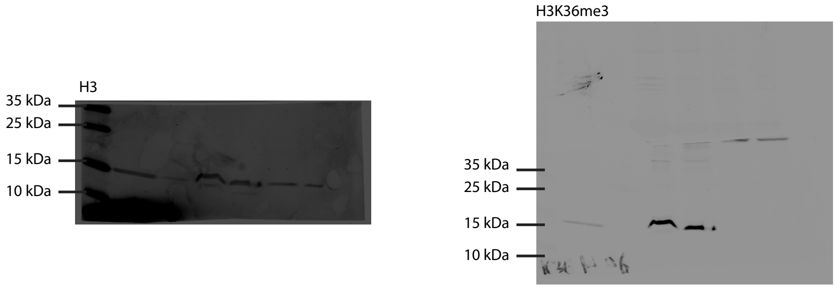

Supplementary Figure 9: Uncropped blots for Figure 5

Figure 5B

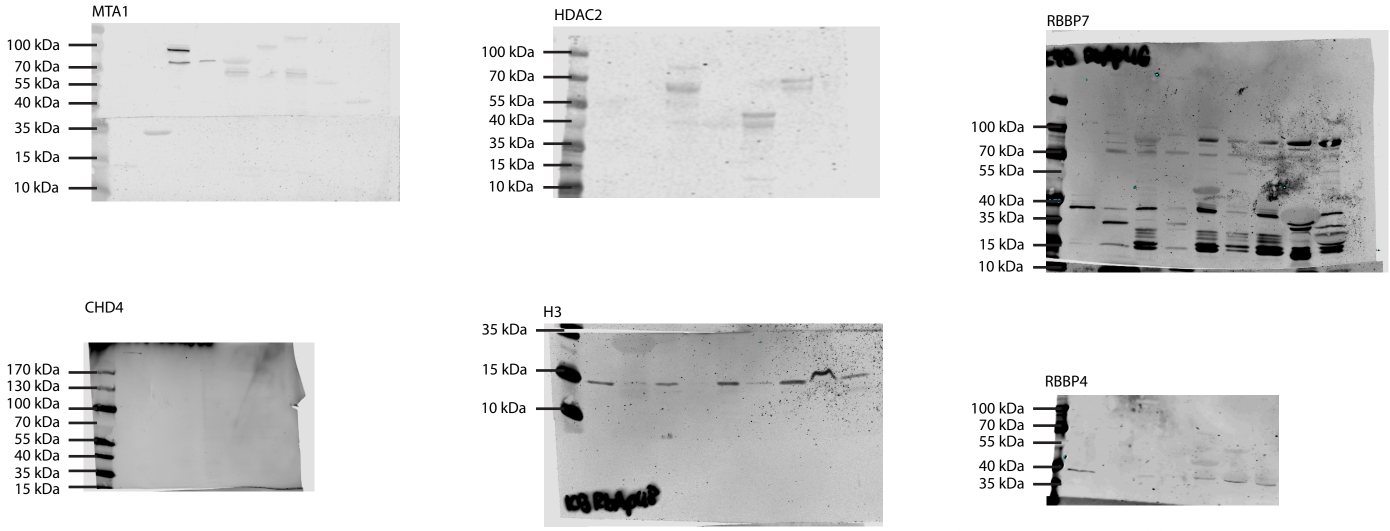


Figure 5D

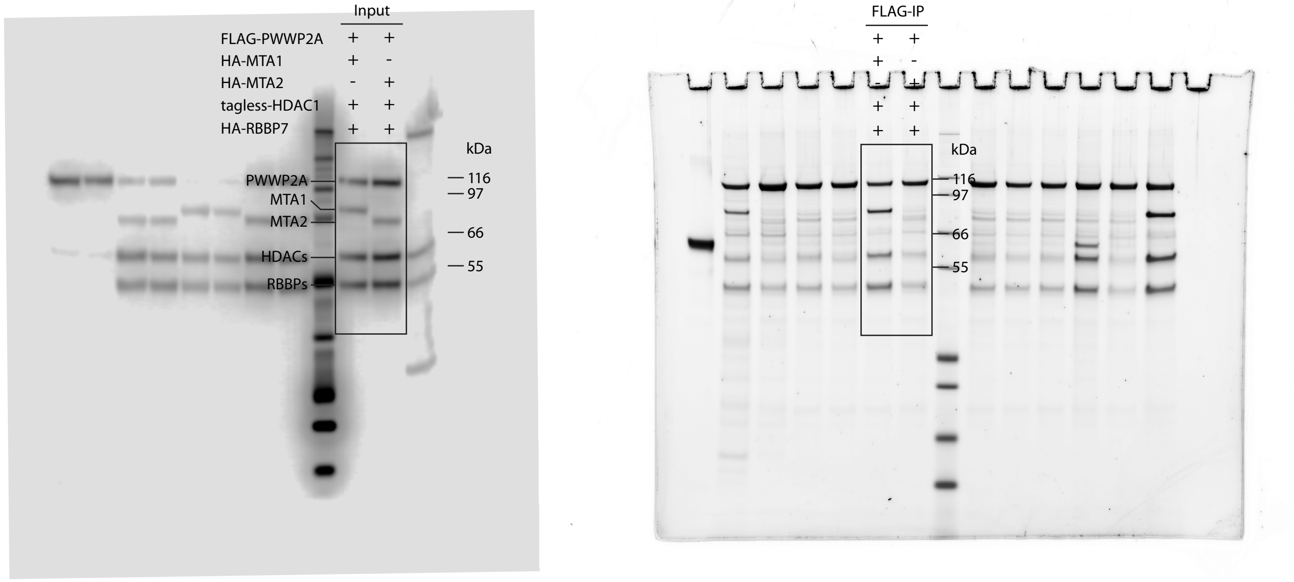


Figure 5E

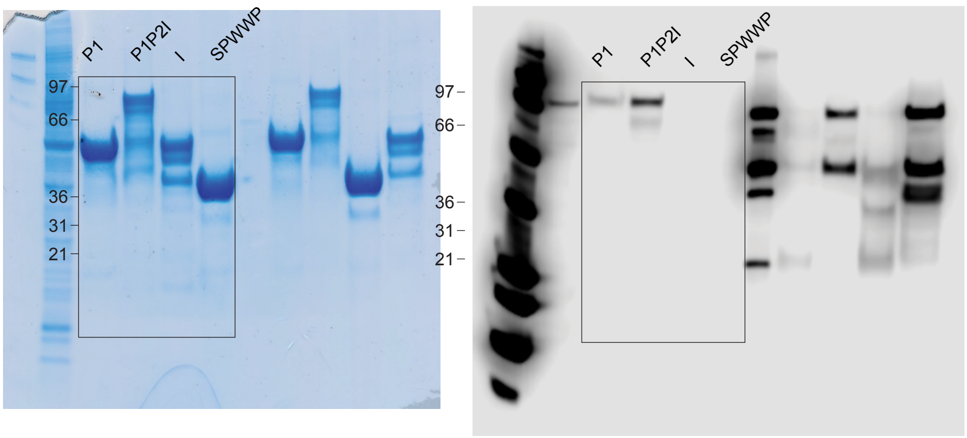

Supplement: Supplementary file 1 — Supplementary Information [file 41467_2018_6665_MOESM1_ESM.pdf]
