# Supplementary material for: PWWP2A binds distinct chromatin moieties and interacts with an MTA1-specific core NuRD complex
Source: Nat Commun. 2018 Oct 16;9:4300. doi: 10.1038/s41467-018-06665-5 (PMC6191444; doi:10.1038/s41467-018-06665-5)
Supplement: Supplementary file 3 — Description of Additional Supplementary Files [file 41467_2018_6665_MOESM3_ESM.pdf]

Description of Additional Supplementary Files

File Name: Supplementary Data 1
Description: List of precursor masses for specified histone modifications and their respective processing parameters for PRM analysis.

File Name: Supplementary Data 2
Description: List of identified proteins of replicate 1 (Supplementary Data 2) and replicate 2 (Supplementary Data 3) of GFPPWWP2A mononucleosome IPs in label-free MS-based proteomics after MaxQuant analysis.

File Name: Supplementary Data 3
Description: List of identified proteins of replicate 1 (Supplementary Data 2) and replicate 2 (Supplementary Data 3) of GFPPWWP2A mononucleosome IPs in label-free MS-based proteomics after MaxQuant analysis.
